# Therapeutic drug monitoring of immunotherapies with novel Affimer–NanoBiT sensor construct†

**DOI:** 10.1039/d3sd00126a

**Published:** 2023-11-01

**Authors:** Emma Campbell, Hope Adamson, Timothy Luxton, Christian Tiede, Christoph Wälti, Darren C. Tomlinson, Lars J. C. Jeuken

**Affiliations:** a School of Biomedical Science, University of Leeds Leeds LS2 9JT UK; b Astbury Centre for Structural Molecular Biology, University of Leeds LS2 9JT UK; c School of Molecular and Cellular Biology, University of Leeds Leeds LS2 9JT UK; d School of Electronic and Electrical Engineering, University of Leeds LS2 9JT UK; e Leiden Institute of Chemistry, Leiden University PO Box 9502 2300 RA Leiden The Netherlands L.J.C.Jeuken@lic.leidenuniv.nl

## Abstract

Concentration–therapeutic efficacy relationships have been observed for several therapeutic monoclonal antibodies (TmAb), where low circulating levels can result in ineffective treatment and high concentrations can cause adverse reactions. Rapid therapeutic drug monitoring (TDM) of TmAb drugs would provide the opportunity to adjust an individual patient's dosing regimen to improve treatment results. However, TDM for immunotherapies is currently limited to centralised testing methods with long sample-collection to result timeframes. Here, we show four point-of-care (PoC) TmAb biosensors by combining anti-idiotypic Affimer proteins and NanoBiT split luciferase technology at a molecular level to provide a platform for rapid quantification (<10 minutes) for four clinically relevant TmAb (rituximab, adalimumab, ipilimumab and trastuzumab). The rituximab sensor performed best with 4 pM limit of detection (LoD) and a quantifiable range between 8 pM–2 nM with neglectable matrix effects in serum up to 1%. After dilution of serum samples, the resulting quantifiable range for all four sensors falls within the clinically relevant range and compares favourably with the sensitivity and/or time-to-result of current ELISA standards. Further development of these sensors into a PoC test may improve treatment outcome and quality of life for patients receiving immunotherapy.

## Abstract 2

An engineered biosensor merges a split luciferase with Affimer proteins to detect pM concentrations of four therapeutic monoclonal antibodies in serum. A 10 minute run time suggests it could be a promising point-of-care test for drug monitoring.

## Graphical abstract

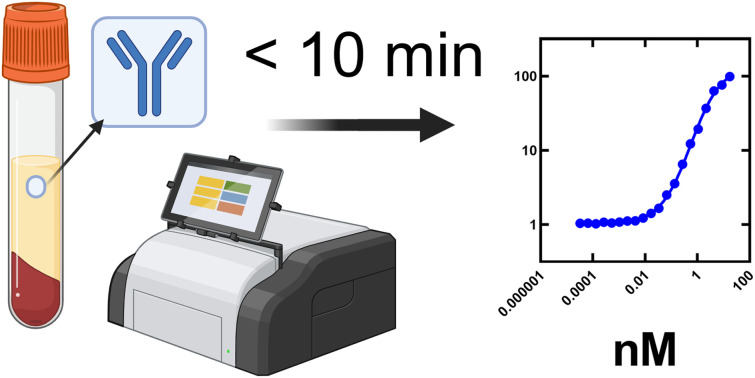

## Introduction

Monoclonal antibodies (mAb) are valuable therapeutic agents with widely employed immunotherapies for autoimmune disorder management and cancer treatment.^1,2^ Therapeutic monoclonal antibody (TmAb) treatments are predominantly immunosuppressive or immune targeting agents,^3,4^ and although TmAbs are generally well tolerated, there have been instances of severe adverse reactions (SARs) due to their promiscuous pharmacological profiles.^5–10^ Moreover, an association between inadequate serum mAb concentration and lack of therapeutic response, alongside interpatient variability of TmAb pharmacokinetics suggest that regular therapeutic drug monitoring (TDM) would be beneficial.^11^ Regular monitoring of ipilimumab trough concentrations between dosages has positively impacted the survival rate of metastatic melanoma patients by preventing drug concentrations from dropping below the minimum effective concentration.^12^ Similarly, maintaining a trough concentration >1.5 μg mL^−1^ of TmAb therapy in Crohn's disease management can improve the remission rate of patients by 76%.^13^

The current TDM methods in use suffer from lengthy processes and require large laboratory-based equipment operated by trained personnel, making them unsuitable for rapid dose adjustment.^14,15^ The introduction of a rapid point-of-care (PoC) dose monitoring platform for TmAbs provides a feedback mechanism so drug concentrations can be maintained within the therapeutic range with the potential of improving treatment efficacy and patient quality of life.^16^ To overcome the lengthy processes (≫ hour) of detection methods like enzyme linked immunoassays (ELISA), homogenous immunoassays, also known as mix-and-read assays, are being developed for diagnostic applications.^17–22^ These simple assays forgo the need for immobilisation or wash steps, making them ideal candidates for implementation into PoC settings.

One homogenous assay method is the split enzyme system, commonly referred to as a proximity switch.^23^ These systems rely on splitting a reporter, usually an enzyme, into two inactive fragments which produce no signal individually or when mixed at concentrations well below the binding affinity of the two fragments.^24–26^ The inactive, split enzyme fragments are each genetically or chemically fused to a recognition element that bind non-overlapping epitopes of the target analyte. Binding of the recognition elements co-localises the two inactive reporter fragments, increasing their effective concentration and prompting re-assembly of the active enzyme, the activity of which can be measured.^17,18,22,27^

**Fig. 1:**
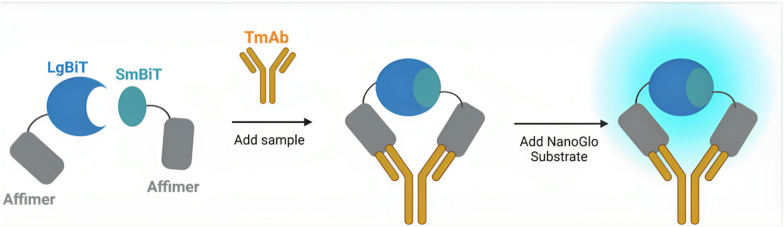
Schematic diagram of Affimer–NanoBiT mechanism of sensing monoclonal antibodies. The luciferase fragments LgBiT and SmBiT101 are attached to the binding reagents *via* short GSG linkers to create two separate sensor components. Antibody binding colocalizes the LgBiT and SmBiT, promoting reconstitution of the enzyme and bioluminescence upon addition of Nano-Glo substrate.

Here, we describe the development of a homogenous, split enzyme assay for the detection of four routinely used TmAb treatments (adalimumab, ipilimumab, rituximab and trastuzumab). The sensor combines Affimer proteins as recognition elements with an established split-luciferase, NanoLuc® Binary Technology (NanoBiT).^17,18,23^ Affimers are non-immunoglobulin-binding proteins and offer the benefit of being small, stable, and easily expressed as recombinant proteins when fused genetically to split-enzyme fragments.^17,20,28,29^ TmAbs, like all mammalian monoclonal antibodies, contain two identical variable regions and thus only one Affimer needs to be selected, as two copies can be used to target non-overlapping epitopes (Fig. 1). With previous work supporting the NanoBiT system as a method of detecting proteins in biological fluids,^17^ we expected our Affimer–NanoBiT system to provide a rapid and sensitive alternative for TDM of TmAb levels that could be implemented into a PoC setting.

## Experimental methods

### Sensor cloning

The DNA and primers (Integrated DNA Technologies) used in this method are detailed in the “DNA and protein sequences” and “Tables of primers” sections (ESI†), respectively. All sensor constructs were generated in a pET28a vector containing NheI, NotI, SpeI and SalI restriction sites between the NcoI and XhoI sites of the vector, with an in frame 6×His-tag sequence and stop-codon following XhoI. Sequential restriction enzyme cloning was used to insert DNA encoding LgBiT, SmBiT (101) or Affimer sequences between NheI/NotI and SpeI/SalI and a (GSG)_7_ linker sequence between NotI and SpeI. The vector was digested with appropriate restriction enzymes (NEB), dephosphorylated with antarctic phosphatase (NEB), separated on an agarose gel, and then purified. All DNA was purified using the Illustra GFX PCR DNA and Gel Band Purification kit (GE Healthcare). The synthetic DNA encoding LgBiT was purchased from Genscript in pUC57 vector. Affimers were encoded in a previously described pEtLECTRA vector.^28^ This insert DNA was PCR amplified with primers encoding appropriate restriction sites, then treated with DpnI (NEB) to remove parental vector DNA. Insert DNA encoding SmBiT101 and (GSG)_7_ linker sequences were generated by PCR of overlapping primers encoding appropriate restriction sites. Amplified insert DNA was purified, digested with appropriate restriction enzymes and then re-purified. The digested vector and insert were ligated with T4 DNA ligase (NEB) and transformed into *E. coli* XL-1 competent cells (Agilent Technologies). Plasmid DNA was purified using the ChargeSwitch Pro Plasmid Miniprep kit (Invitrogen) and successful generation of constructs was confirmed by sequencing (Genewiz) with T7/T7 term primers.

### Sensor expression and purification

The pET28a vectors with sensor constructs were transformed into *E. coli* BL21* (DE3) cells. A 1 mL starter culture was added to 50 mL LB media (with 50 μg mL^−1^ kanamycin) and grown at 37 °C, 220 RPM before induction at OD_600_*ca.* 0.6 with 0.3 mM isopropyl-β-d-thiogalactoside (IPTG) and grown overnight at 16 °C, 180 RPM. Cells were harvested at 4000×*g* for *ca.* 20 min, resuspended in 4 mL lysis buffer (pH 7.4, 50 mM Tris, 300 mM NaCl, 10 mM imidazole, 0.1 mg mL^−1^ lysozyme, 1× cOmplete EDTA-free protease inhibitor (Merck), 0.001% v/v benzonase nuclease (Merck)) and incubated on a roller mixer for 1 hour at 4 °C. Cells were lysed by sonication (UP50H, Hielscher) for 2 min (5 s on/5 s off) at 100% amplitude then pelleted at 17 000×*g* for 20 min. The supernatant was added to 250 μL Super Co-NTA resin (Generon) that had been pre-equilibrated with wash buffer (pH 7.4, 50 mM Tris, 300 mM NaCl, 10 mM imidazole) and was then incubated on a roller mixer for 1 hour at 4 °C. The resin was washed thrice with 5 mL wash buffer and protein eluted with 3 × 0.5 mL elution buffer (pH 7.4, 50 mM Tris, 300 mM NaCl, 300 mM imidazole). Pure fractions (as assessed by SDS-PAGE) were buffer exchanged into storage buffer (50 mM Tris, 150 mM NaCl, pH 7.4) using Zeba spin desalting columns (ThermoFisher). Protein concentration was determined by BCA assay and aliquots stored at −80 °C.

## Sensor characterisation

### Target mAbs

Biosimilars of each of the target mAbs were purchased from InvivoGen; rituximab (anti-hCD20-hIgG4 S228P), adalimumab (anti-hTNF-α-hIgG1), trastuzumab (anti-HER2-hIgG4 S228P) and ipilimumab (anti-hCTLA4-hIgG1).

### NanoBiT assay (in buffer)

Assays were performed in PBSB (pH 7.4, PBS + 1 mg mL^−1^ BSA) dilution buffer. 10 μL LgBiT sensor (5× final conc.), 10 μL SmBiT sensor (5× final conc.) and 5 μL mAb (InvivoGen) (10× final conc.) were added to a well of a white no-bind 384-well plate (Corning) and incubated for 30 min, 25 °C, shaking. Then 25 μL of 1 : 500 Nano-Glo was added to give a final dilution of 1 : 1000. Luminescence was read (500 ms integration) on a Tecan Spark plate reader. Parameter changes for optimisation are depicted in the Results section or ESI.†

### NanoBiT assay (with serum)

For experiments in 0–1% serum, assays were performed in PBSB (pH 7.4, PBS + 1 mg mL^−1^ BSA) dilution buffer. 10 μL 10 nM LgBiT + 10 nM SmBiT in PBSB (2 nM each final conc.) and 5 μL mAb (InvivoGen; 10× final conc.) in PBSB were added to 10 μL human serum (pooled human serum, Clinical Trials Laboratory Services) at 5× the final concentration (in PBSB) in a white no-bind 384-well plate (Corning) and incubated for 30 min, 25 °C, shaking.

For experiments in 5 and 50% serum, assays were performed by first preparing stock solutions (LgBiT, SmBiT, mAb) in either 10% human serum with PBSB or 100% human serum. 10 μL of 10 nM LgBiT sensor (5× final conc.), 10 μL of 10 nM SmBiT sensor (5× final conc.) and 5 μL mAb (InvivoGen; various concentrations at 10x the final conc.) were added together in a well of a white no-bind 384-well plate (Corning) and incubated for 30 min, 25 °C, shaking.

For all assays, bioluminescence was initiated by addition of 25 μL of 1 : 500 Nano-Glo to give a final dilution of 1 : 1000. Luminescence was read (500 ms integration) on a Tecan Spark plate reader. Parameter changes for optimisation are depicted in the Results section.

### Data analysis

All data analysis was performed using GraphPad, Prism software version 9.0. Graphs presented as fold gain refer to [RLU (*X* nM TmAb)/RLU (0 nM TmAb)]. Optimisation experiments presented as the mean of at least 2 repeats ± the standard error of the mean (SEM) unless specified otherwise. Intra- and inter-assays presented as *n* = 3 or *N* = 3 ± SEM. Statistical significance was determined as *P* < 0.05, using one-tailed homoscedastic *t*-tests.

Limit of detection (LoD) was defined as the lowest concentration of analyte that produced a reading above a minimal value (RLU_min_):RLU_min_ = RLU_blank_ + 1.645(SD_blank_) + 1.645(SD_low conc._)in which RLU_blank_ is the average reading without analyte, SD_blank_ = the standard deviation of samples without analyte and SD_low conc._ = the standard deviation of samples with analyte at the lowest concentration for which a signal above baseline is produced.^30^

Accuracy of assay performance was measured as percentage recovery:% recovery = (mean interpolated concentration/nominal concentration) × 100%and precision measured as percentage coefficient variation:% CV = (SD interpolated concentration/mean interpolated concentration) × 100%

## Results

### Selection and characterisation of binding proteins

An Affimer reagent phage display library was screened against target antibodies trastuzumab, rituximab, adalimumab, and ipilimumab, as described previously.^28,31^ Briefly, the binding reagents selected for in three rounds of phage panning were subject to ELISA validation, and a lead candidate was chosen for each target TmAb and further characterised.^28^ Surface plasmon resonance (SPR) was used to determine the affinity of each anti-idiotypic Affimer protein. TmAb biosimilars were covalently immobilised onto an SPR chip and titrated with serial dilutions of respective Affimer reagents. Nanomolar affinities were confirmed for all antibody–Affimer reagent complexes (Table 1).

**Table 1:** *K*_D_ values calculated from evaluation of Langmuir model fits of SPR curves (Fig. S1†). All anti-idiotypic Affimer proteins have nM affinity for their respective TmAb analytes and are within ∼12-fold of one another. *K*_D_ values are presented as a mean of three replicates ± SEM. (Aff–Ipi – ipilimumab *n* = 2)

	*K*_D_ (nM)	SEM (nM)
Aff–Trast – trastuzumab	0.75	±0.12
Aff–Ipi – ipilimumab	7.8	±0.8
Aff–Ada – adalimumab	9.8	±2.5
Aff–Rit – rituximab	4.0	±0.75

## Split luciferase sensor development

Luciferase enzymes are commonly used in split enzyme proximity switches with successful recombination seen from multiple luciferases.^32–34^ An engineered catalytic subunit of a luciferase from the deep-sea shrimp (*Oplophorus gracilirostris*) was isolated and termed NanoLuc.^23,35^ The small size and high stability of this luciferase subunit allows for splitting of NanoLuc into two inactive fragments that recover their enzymatic activity with reassembly, known as the NanoBiT system.^17,18,23^ Here we used the 18 kDa LgBiT and 11 amino acid SmBiT101 peptide (VTGYRLFEKES) as the reporter fragments. The LgBiT and SmBiT101 fragments were genetically fused to either the N-terminus or C-terminus of the anti-idiotypic binding reagents to produce four pair combinations for each TmAb. A (GSG)_7_ peptide linker was inserted between the NanoBiT fragment and the binding reagent. We will use one letter codes for the LgBiT (**L**) and SmBiT101 (**S**) fragments, as well as for the four Affimers raise against the four TmAbs (adalimumab, **A**; ipilimumab, **I**; rituximab, **R**; and trastuzumab, **T**). The order of the one-letter codes represents N-terminal *vs.* C-terminal constructs with, for instance, **L**–**A** denoting an Affimer against adalimumab with an N-terminal LgBiT fragment, connected *via* a (GSG)_7_ linker. Full protein sequences are given in the supplementary information. All 16 possible combinations were constructed and expressed in *E. coli* and purified *via* a C-terminal 6×His-tag (Fig. S2†).

**Fig. 2:**
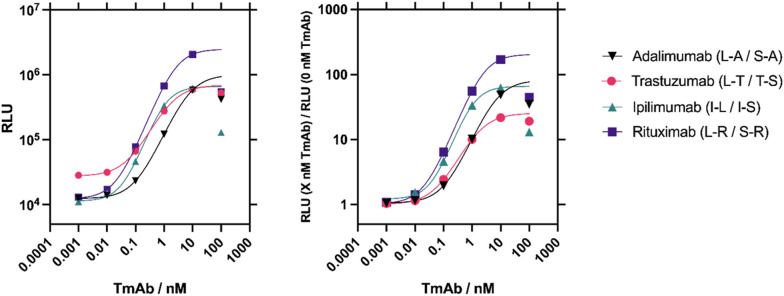
Four sensor combinations were trialled for each target TmAb and the optimal pair chosen for each. Raw luminescence data (left) and fold gain data (right) from NanoBiT assays performed on the best performing LgBiT/SmBiT101 pairs selected for each target TmAb. Sigmoidal, 4 parameter logistic (4PL) fits were used on data points up to concentrations of 10 nM. Due to the evident hook effect at concentrations of 100 nM, these data points were not included in the fit and the curve was extrapolated for these points. *n* = 1.

To determine the optimal sensor pair combinations for TmAb quantification, 2 nM of each sensor pair was incubated with a range of 0–100 nM respective TmAb, and after addition of substrate, bioluminescence was measured (Fig. S3†). Sensor pairs displayed responses ranging from 5-fold to 170-fold gain in bioluminescence. An obvious hook effect is observed at >10 nM TmAb titres, which is likely due to the excess of TmAb relative to the split enzyme–Affimer constructs (2 nM). A 2 nM component concentration was chosen as a starting point for initial experiments due to previous success when detecting other biomarkers.^17^ When TmAb is in excess, it is stochastically likely only one Affimer binds to each TmAb (Fig. 2). Even without excess it is possible that two SmBiT or two LgBiT fragments bind to the same TmAb, which should reduce the ensemble signal. Still, the binding affinity between the SmBiT101 and LgBiT fragments will provide a slight thermodynamic advantage to the formation of a SmBiT101–LgBiT–TmAb complex.

The best sensitivity and activity fold gain was seen by the **L**–**R**/**S**–**R** sensor pair against rituximab and thus this rituximab sensor was chosen to further optimise the assay conditions (Fig. S4†). First, the ideal sensor concentration was determined (Fig. S4A†). Higher concentrations of LgBiT and SmBiT components resulted in a larger relative light unit (RLU) response but also contributed to much higher background complementation. In contrast, the lowest concentration had much lower background, but reduced the maximum analyte-driven reconstitution. To account for both extremes a mid-range concentration of 2 nM for each sensor component was selected to maximise the ratio of analyte-induced to background bioluminescence signal.

The time-to-result is key for the successful development of a point-of-care (PoC) dose monitoring platform. The shortest, optimal assay time was thus determined at various substrate concentrations (Fig. S4B–E†). Increasing substrate concentration increases the RLU, but also the time required before maximum activity is observed (Fig. S4E†). After an initial rise between 2 to 5 min, the signals slowly decayed over time (Fig. S4C and E†). When analysed in terms of fold-gain, differences were significant but very small (Fig. S4B and D†). To optimise the assay for speed and sensitivity, bioluminescence was recorded 2 minutes after addition of the substrate (Nano-Glo) at a dilution factor of 1 : 1000 in the final assay.

Another aspect of the NanoBiT assay that could be optimised was the incubation time between the Affimer constructs and TmAb (at 25 °C) prior to substrate addition (Fig. S4F†). Maximum activity was already observed within 2.5 min, with the signal remaining stable thereafter. Therefore, and to allow time to prepare multiple tests at once, a 25 °C incubation step of ≥2.5 minutes should be used before adding the substrate. Importantly, when all reagents are prepared in advance, the time-to-results of the optimised assay is under 10 minutes.

The functionality of the NanoBiT sensor assay in pooled human serum was tested to establish its feasibility as a PoC TDM test. No significant difference in maximum fold gain of bioluminescence signal was detected in up to 1% pooled human serum (*P* > 0.05) (Fig. S4G†). However, sensor activity was significantly diminished in 50% serum. We optimised the serum sample dilution so that the therapeutic range of rituximab was within the linear range of the interpolated curve. The therapeutic range of rituximab is approximately 150–500 nM.^36^ In 0.1% serum the LOD of the rituximab NanoBiT assay is ∼8 pM (see below), therefore, a 1000× dilution of serum samples would result in TmAb concentrations quantifiable in this assay.

**Fig. 3:**
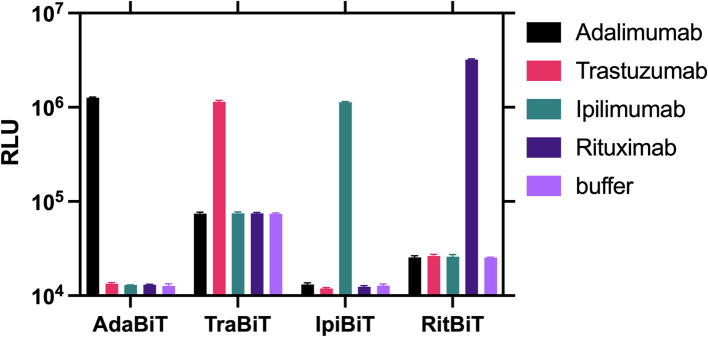
The four best performing sensor combinations for each target TmAb are highly specific for their respective TmAb. Raw luminescence from NanoBiT assays performed on the selected optimal LgBiT/SmBiT101 pairs for all four target TmAb. A final concentration of 2 nM LgBiT and SmBiT were used with 5 μg mL^−1^ (35 nM) of each TmAb. Data are presented as a mean of 3 repeats with error bars representing standard deviation.

Before applying the optimised protocol to the other three sensor pairs, the specificity of each sensor was tested against the four TmAbs and blank buffer as a measurement of bioluminescence (Fig. 3). All sensors are highly specific to their target TmAb, with no response to non-specific TmAb targets significantly higher than buffer (*P* > 0.05).

**Fig. 4:**
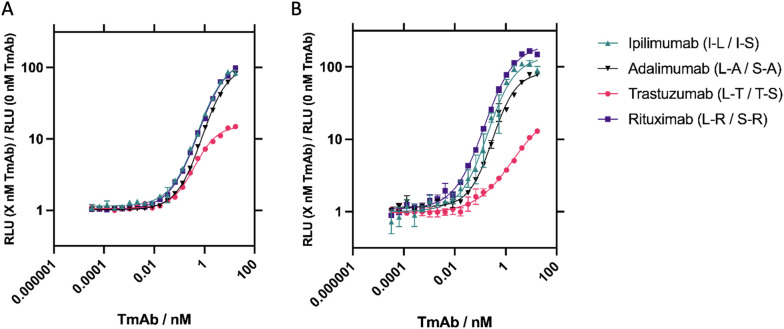
All four TmAb NanoBiT sensors have similar quantifiable ranges. Data on all sensors at 2 nM in response to increasing concentrations of their respective TmAb. Assays were performed in 0.1% pooled human serum, incubated for 5 minutes shaking at 25 °C, and RLU measurements taken after 2 minutes. Standard curves were interpolated for all four sensors using sigmoidal, 4 parameter logistic (4PL) fits. A *n* = 3, performed on the same day with the same reagents. B *N* = 3, performed on separate days, using fresh reagents. Data is presented as fold gain activity and error bars represent ± SEM.

Finally, performance and intra-assay and inter-assay variability were determined for the four best-performing sensor combinations for each of the TmAb. In the final optimised assay, 10 μL of 10 nM SmBiT101 and 10 nM LgBiT (2 nM final concentration) was incubated with 10 μL 0.5% pooled human serum (0.1% final concentration) and 5 μL of varying TmAb concentration from 0.005–2500 ng mL^−1^ (∼16 fM–17 nM) for 5 minutes shaking at 25 °C. 25 μL 1 : 500 dilution of Nano-Glo substrate (final dilution 1 : 1000) was then added and bioluminescence readings were taken after 2 minutes. For intra-assay assessment, measurements were taken as *n* = 3 performed on the same plate (Fig. 4 and S5A†) and to determine inter-assay variation, 3 independent measurements were taken on separate dates for each target analyte, *N* = 3 (Fig. 4 and S5B†). Concentrations from 0.005–2500 ng mL^−1^ (∼16 fM–17 nM) were used to create a standard curve and concentrations interpolated back to determine percentage recovery and percentage coefficient of variance (CV) to assess accuracy and precision respectively. Quantifiable ranges were subsequently calculated by percentage recovery between 80% and 111% and percentage CV < 20% (or <25% at limit of quantification)^37^(Table 2).

**Table 2:** Sensitivity (LoD), accuracy (% recovery), and precision (% CV) of TmAb NanoBiT assays, as determined from raw bioluminescence (Fig. 4A, S5A and C†), or fold gain data (Fig. 4B, S5B and D†), to define a quantifiable range

Sensor target	Feature	Intra-assay raw data	Intra-assay fold gain	Inter-assay raw data	Inter-assay fold gain
Rituximab (**L**–**R**/**S**–**R**)	Sensitivity (LoD)	8 pM	8 pM	17 pM	4 pM
	Quantifiable range	8 pM–17 nM	8 pM–17 nM	17 pM–269 pM	8 pM–2 nM
	% recovery	82–110%	82–110%	95–109%	96–106%
	% CV	1–15%	0.5–18%	5–18%	5–24%^a^
Trastuzumab (**L**–**T**/**T**–**S**)	Sensitivity (LoD)	17 pM	17 pM	269 pM	67 pM
	Quantifiable range	33 pM–2 nM	33 pM–2 nM	540 pM–5 nM^b^	269 pM–17 nM
	% recovery	82–110%	82–110%	88–109%	88–110%
	% CV	1–10%	1–10%	53–54%	3–17%
Ipilimumab (**I**–**L**/**I**–**S**)	Sensitivity (LoD)	8 pM	134 pM	33 pM	17 pM
	Quantifiable range	134 pM–2 nM	134 pM–2 nM	33 pM–269 pM^b^	33 pM–5 nM
	% recovery	91–101%	93–110%	92–111%	86–111%
	% CV	1–10%	1–6%	15–61%	1–20%
Adalimumab (**L**–**A**/**S**–**A**)	Sensitivity (LoD)	8 pM	8 pM	67 pM	33 pM
	Quantifiable range	33 pM–4 nM	33 pM–8 nM	67 pM–2 nM	67 pM–5 nM
	% recovery	92–105%	92–108%	88–109%	81–109%
	% CV	1–5%	1–9%	0.5–18%	1–19%

a % CV precision metrics >20% only at the limit of quantification.

b Quantifiable range based on % recovery only.

## Discussion

TDM is a valuable tool when using immunotherapies or immunosuppressive agents which can improve patient outcomes.^38^ TDM requires the measurement of drug concentrations in blood samples immediately before and immediately after each cyclical dosage to determine peak and trough concentrations.^39^ Knowledge of these values provides the possibility of immediate dose adjustment for an individual, as well as providing valuable data on the pharmacokinetic (PK) and pharmacodynamic (PD) profile of the drug in question. These parameters can inform clinical staff on how to maintain an effective circulating drug concentration. Currently, TDM is performed on rituximab, ipilimumab, adalimumab and trastuzumab in an ELISA format,^39–43^ but these require long wash and incubation steps leading to slow time-to-result thus limiting the effect of TDM.

The TmAb NanoBiT assays developed here measured TmAb drug levels accurately and precisely, down to reported trough concentrations for all four therapeutics with a time-to-result of under 10 minutes. Intra-assay variability for the sensors was low, with high sensitivity, accuracy and precision when detecting TmAb in 0.1% pooled human serum. When assessing inter-assay variability, the sensors against rituximab and adalimumab exhibited high sensitivity, accuracy, and precision (Table 2). Sensors against trastuzumab and ipilimumab showed high sensitivity and accuracy, the precision of these sensors was improved by analysing fold-gain data. Normalisation of the data against blank measurements to give fold-gain values recovered the % CV values of these sensors to provide a wider range of quantification. Substantial intra- and inter-assay variability is commonly seen in binding assays when using raw data due to a range of condition variations.^44^ Utilising blank or background signal to produce normalised ratio values works as a local control which limits confounding factors and specimen-to-specimen variability, giving more consistent results.^45^ The use of fold-gain data improved LoD and the upper limit of quantification (ULOQ) (Table 2).

Bioluminescent reporters are appealing for PoC applications as they do not require external excitation and have been used in the development of homogenous assays for the detection of mAbs.^46–48^ One such example is the LUMinescent AntiBody Sensor (LUMABS) which has been adapted for quantification of trastuzumab^46^ (Table S1†). Similar to our design, LUMABS is a homogenous assay that does not require wash steps, however, requires 2.5 hours of incubation prior to signal output. The sensitivity of this system for trastuzumab detection is 5× lower than our reported sensitivity, however, due to the ratiometric nature of the LUMABS signal, undiluted serum samples can be used. Including the time taken to dilute serum, our NanoBiT sensor would still be 10× faster than LUMABS. Paper-based adaptions of LUMABS for mAb detection have decreased the time-to-result, however this has not yet been used for quantification of TmAbs.^48^ LUMABS use antibody epitopes as recognition elements which are highly selective but require prior knowledge of epitope binding. Our use of Affimer proteins, which can be selected through phage display, broadens the scope of targets the NanoBiT system can be adapted to. As an alternative to epitopes, *de novo* proteins have since been implemented into LUMABS.^47^

The sensitivity of the NanoBiT biosensor would allow for substantial dilution of patient samples to keep analyte concentrations within the range of quantification. Trough concentrations of rituximab are reported between 8–400 nM,^49^ with circulating levels <84 nM after the first cycle of treatment associated with poor treatment outcome for follicular lymphoma patients.^39^ With a 1000× dilution of serum samples, the minimal effective concentration (*C*_min_) falls within our NanoBit assay range of quantification. The current standard for serum rituximab measurements is an ELISA with a limit of detection (LoD) of 3 ng mL^−1^ (≈31 pM) in commercial kits using a 1000× diluted serum sample (Table S1†). Our LoD (4 pM) and LLOQ (8 pM) are thus in the same range with the significant advantage of a shorter timeframe, with sample collection to result possible within 10 minutes if reagents are preprepared. Similarly, the quantifiable range for our ipilimumab and trastuzumab sensors cover the reported trough concentration range and *C*_min_ values for both drug therapies,^43,50^ while the LoD and LLOQ are in the same range as commercially available ELISA kits (Table S1†). The sensitivity of these biosensors also compares favourably to other split luciferase assays developed for monoclonal antibody detection.^51,52^ Besides the improvement in time-to-result, homogenous assays as employed here do not require any wash steps and are thus ideally suited to be developed into bedside or PoC sensors.

During the maintenance period (between dosing), adalimumab concentrations >50 nM in paediatric cases and >35 nM in adult cases are associated with irritable bowel disease (IBD) and Crohn's disease remission.^42,53,54^ Currently our adalimumab sensor has a LLOQ of 67 pM and after serum dilution samples with adalimumab concentrations <50 nM would be just outside of the quantifiable range. During the optimisation stage of this work there was minimal difference in assay activity in up to 1% serum. To this end, higher serum concentrations could be trialled to obtained lower LLOQ for the adalimumab sensor. Assay optimisation was performed on the rituximab sensor **L**–**R**/**S**–**R**, so it is unsurprising that this was the best-performing sensor. Performance of the adalimumab sensor could possibly be further improved with individual optimisation.

Biosensor performance has so far been validated by spiking pooled human serum, further assessment using patient samples would provide better insight into the real-world applications. The 1 : 1000 dilution factor applied to spiked samples to maintain TmAb concentrations within the quantifiable range, would suggest that any matrix effects would be negligible. However, there are elements within patient samples that are not represented by pooled serum from healthy individuals. Tumour shedding in advanced HER2+ breast cancer produces circulating exosomes containing HER2 extracellular domain (ECD) which can bind to and reduce the pharmacological effect of trastuzumab.^55^ Biological treatments, such as immunotherapies, carry the risk of developing anti-drug antibodies (ADAs). For patients treated with trastuzumab, ipilimumab and rituximab, the risk of ADA formation is low,^56^ however, the prevalence of ADAs in adalimumab treatment is much higher.^57^ HER2 ECD and ADAs typically interact with the variable regions of TmAb, with inhibitory effects. The remaining free (pharmacologically active) TmAb concentration is the target of TDM.^58^ We can speculate, with recognition elements targeting the variable regions of TmAb, that our sensing system would not measure bound, inactive TmAb concentrations. Future testing of our TmAb NanoBiT sensors on samples taken from patients undergoing these treatments would help inform on the applicability as a PoC test.

## Conclusion

In conclusion, a TmAb NanoBiT assays was developed by combining anti-idiotypic Affimer proteins and NanoBiT split luciferase technology to provide a platform for rapid quantification of four immunotherapies. Assay conditions such as incubation time, sensor component and substrate concentration were optimised to develop an assay with a possible time-to-result within 10 minutes. Low pM LoD values and quantifiable ranges that fall within the therapeutic ranges of ipilimumab, rituximab and trastuzumab were determined in 0.1% spiked human serum. The sensors for detection of these three TmAbs had comparable or improved performance metrics to the current ELISA standards. The possibility of a time-to-result within 10 minutes without any wash steps make our sensors an appealing alternative to ELISA detection, with the prospect of implementing into a PoC device in the future.

The concentration-therapeutic efficacy relationship of therapeutic monoclonal antibodies means that serum drug concentrations outside of the therapeutic window can have negative impacts on patient health. TDM for immunotherapies is currently limited by centralised testing methods with long sample-collection to result timeframes. Our TmAb NanoBiT assays with a time-to-result within 10 minutes could thus improve patient welfare by providing the opportunity for rapid, precise dose adjustments to improve treatment outcomes and prevent adverse reactions.

## Author contributions

Emma Campbell: methodology, investigation, analysis, writing – original draft, review & editing, Hope Adamson: conceptualisation, methodology (sensor expression and purification, initial experimentation), Timothy Luxton: methodology (rituximab sensor optimisation), Christian Tiede: methodology (initial Affimer protein screen), Darren C. Tomlinson: methodology, supervision of Affimer protein isolation, Lars J. C. Jeuken: supervision, writing – review & editing, Christoph Wälti: supervision.

## Conflicts of interest

The authors declare the following competing financial interest(s): the Affimer reagents used in this report are owned by the University of Leeds (UoL) but licensed to Avacta Life Sciences. The UoL received royalties from Avacta Life Sciences as part of the license agreement, which is managed by the commercialization team. The authors declare no competing financial interest.

## Supplementary Material

SD-003-D3SD00126A-s001
