# Supplementary material for: Therapeutic drug monitoring of immunotherapies with novel Affimer–NanoBiT sensor construct
Source: Sens Diagn. 2023 Nov 1;3(1):104–11. doi: 10.1039/d3sd00126a (PMC10795742; doi:10.1039/d3sd00126a)
Supplement: SD-003-D3SD00126A-s001 [file SD-003-D3SD00126A-s001.pdf]

Electronic Supplementary Material (ESI) for Sensors & Diagnostics.
This journal is © The Royal Society of Chemistry 2023

Supplementary Information

Therapeutic drug monitoring of immunotherapies with novel Affimer-NanoBiT sensor construct

Emma Campbell[1,2], Hope Adamson[1,2], Timothy Luxton[1,2], Christian Tiede[2,3], Christoph Wälti[4], Darren C Tomlinson[2,3], Lars JC Jeuken[1,2,5,*]

[1] School of Biomedical Science, University of Leeds, Leeds, LS2 9JT, United Kingdom
[2] Astbury Centre for Structural Molecular Biology, University of Leeds, LS2 9JT, United Kingdom
[3] School of Molecular and Cellular Biology, University of Leeds, Leeds, LS2 9JT, United Kingdom
[4] School of Electronic and Electrical Engineering, University of Leeds, LS2 9JT, United Kingdom
[5] Leiden Institute of Chemistry, Leiden University, PO Box 9502, 2300 RA, Leiden, the Netherlands
* Corresponding author: L.J.C.Jeuken@lic.leidenuniv.nl

Contents

Supplementary Methods
Supplementary Figures
DNA and Protein Sequences
Table of Primers

Supplementary Methods

Surface plasmon resonance

Affimer affinities for their TmAb analyte were determined by surface plasmon resonance (SPR) using a BIAcore 3000 (GE Healthcare Europe GmbH). Trastuzumab, ipilimumab, adalimumab and rituximab were covalently immobilized on separate channels of a CM5 sensor chip with amine-coupling chemistry. The chip was activated with 200 mM 1- Ethyl-3-(3-dimethylaminopropyl) carbodiimide (EDC) and 50 mM N-Hydroxysuccinimide (NHS) before target injection under optimised conditions (trastuzumab, 5 µg mL^{-1} in 10 mM acetate pH 5.5; ipilimumab, 5 µg mL^{-1} in 10 mM acetate pH 5.5; rituximab, 5 µg mL^{-1} in 10 mM acetate pH 5.5; adalimumab, 5 µg mL^{-1} in 10 mM acetate pH 4.5). Remaining reactive groups were capped with ethanolamine (1 M, pH = 8.5). Biacore experiments were performed at 25°C in PBST buffer (PBS pH 7.4, containing 150 mM NaCl and 0.2 % Tween 20). Affimers were injected at 1.5625, 3.125, 6.25 and 12.5 nM at a flow rate of 5 µL min^{-1}, followed by 12 minute dissociation. The on- and off- rates and Kd parameters were obtained from a global fit to the SPR curves using a 1:1 langmuir model, using the BIAevaluation software. Quoted Kd values are the mean ± SEM of three replicate runs, unless specified otherwise.

Supplementary Figures

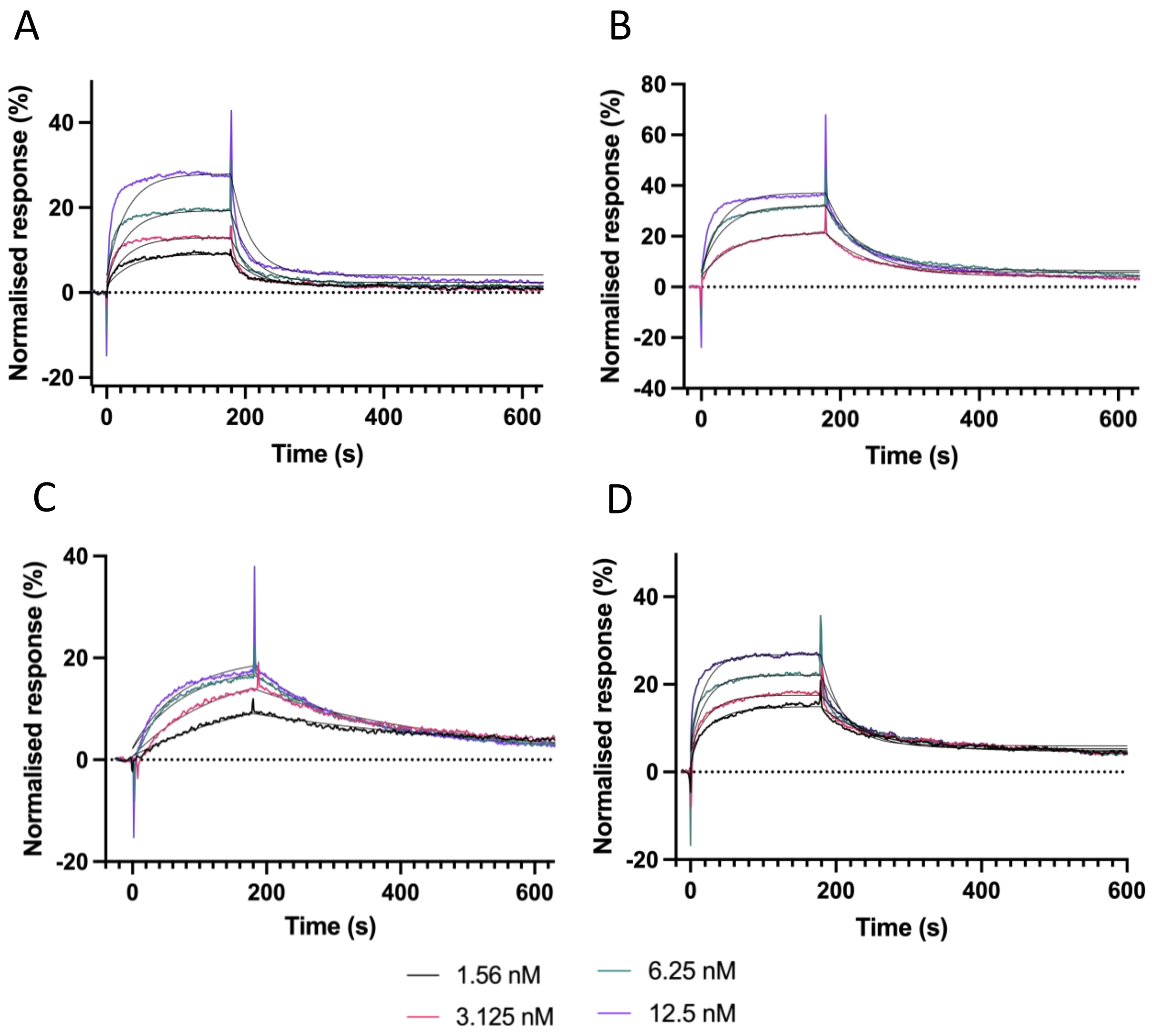

Figure S1. Surface Plasmon Resonance (SPR) curves plotted for each anti-idiotype Affimer to analyse binding to their respective TmAb. *Aff-Ada and Adalimumab (**A**), Aff-Ipi and ipilimumab (**B**), Aff-rit and rituximab (**C**) and Aff-trast and trastuzumab (**D**). SPR plots are shown between TmAb concentrations of 1.56 nM and 12.5 nM. Langmuir model fits are plotted alongside each concentration for each TmAb-Affimer binding study. SPR experiments were conducted in triplicate (duplicate for Aff-Ipi) however only replicate one has been included here. Initial evaluation of SPR data and Langmuir model fits were conducted on BIAevauation software. Raw data for all plots and fits were then extracted from BIAevaluation and plotted on GraphPad to improve visualisation of data.*

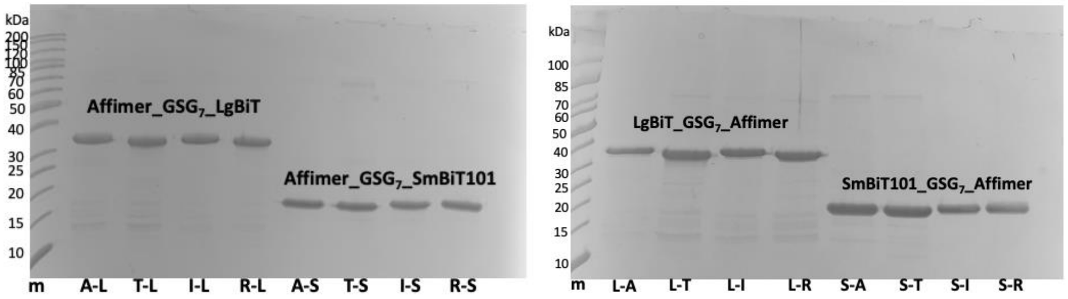

Figure S2. SDS-PAGE of purified Affimer-NanoBiT sensors. Left: *visualisation of anti-ID Affimer NanoBiT construct where the luciferase fragment has been attached to the C-terminus of the Affimer protein with a polypeptide linker.* ***Right:*** *visualisation of anti-ID Affimer NanoBiT construct where the luciferase fragment has been attached to the N-terminus of the Affimer protein with a polypeptide linker.*

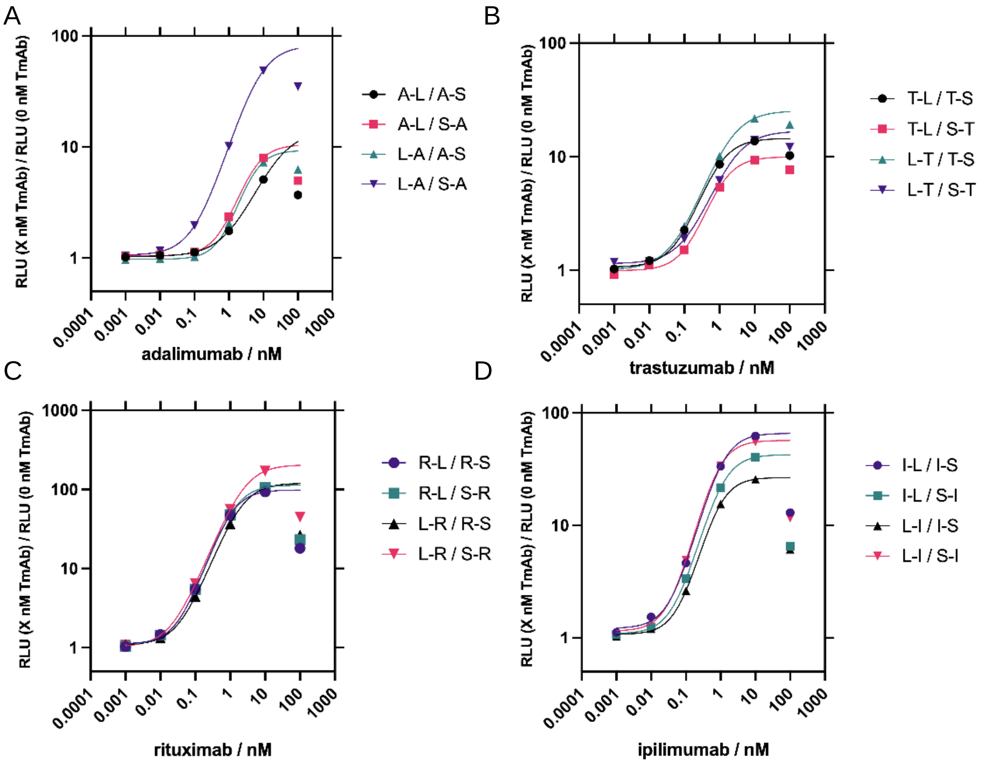

Figure S3. selection of best LgBiT / SmBiT101 NanoBiT sensor combinations for each TmAb target. *A: anti-adalimumab sensor pairs, B: anti-trastuzumab sensor pairs, C: anti-rituximab sensor pairs, D: anti-ipilimumab sensor pairs. 10 µL of each sensor, to a final concentration of 2 nM, were incubated with 5 µL of target TmAb at final concentrations ranging from 0.001 – 100 nM. Plates were incubated at 25°C, shaking for 30 minutes. 25 µL of NanoGlo diluted 1:500 was then added, and luminescence readings taken immediately (t = 0). Sigmoidal, 4 parameter logistic (4PL) fits were used on data points up to X = 10 nM. Due to the evident hook effect at X = 100 nM, these data points were not included in the fit and the curve was extrapolated for these points. n = 1.*

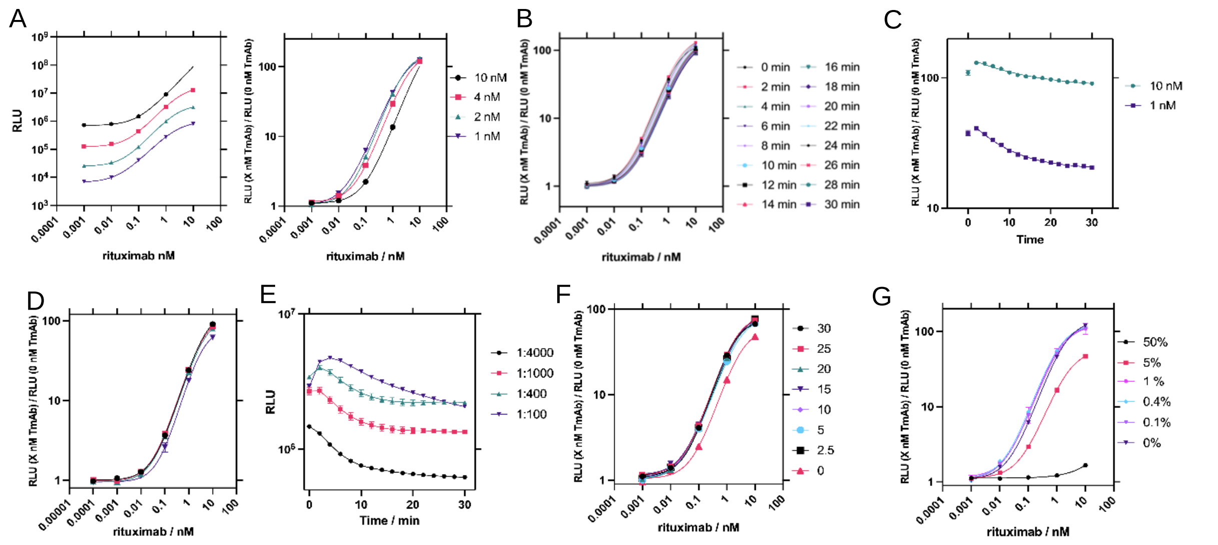

Figure S4. Optimisation of assay to detect therapeutic monoclonal antibodies using rituximab as a template target. A *The higher the concentration of sensor components, the less sensitive the assay. RLU (left) and Fold gain (right) data from NanoBiT assays performed on the selected optimal LgBiT / SmBiT101 pair for rituximab with varying sensor concentrations. Measurements taken 2 minutes after substrate incubation. Sigmoidal, 4 parameter logistic (4PL) fits were used on data points up to X = 10 nM. Due to RLU measurements of the 10 nM sensor pair at X = 10 nM being over the limit of RLU measurement of the Tecan, the fits were extrapolated for these points, but no data points were plotted. n = 2, data is plotted as a mean of 2 repeats with ±SEM, where error bars are not visible, they are within the symbol.* ***B*** *RLU measurements are highest 2 minutes after substrate addition. Fold gain measurements in response to 0.001 – 10 nM rituximab measured every 2 minutes from t = 0 to t = 30 minutes. Sigmoidal, 4 parameter logistic (4PL) fits were used with data points plotted as n = 2 with error bars representing ±SEM.* ***C*** *Luminescence signal decay begins 2 minutes after substrate addition**.** Change in fold-gain luminescence signal of anti-rituximab sensor (L-R/S-R) in response to 10 nM and 1 nM rituximab, plotted against time. RLU readings were taken every 2 minutes from 0-30 minutes post substrate incubation. Non-linear regression curves were fit using a one phase exponential decay equation* $Y = Span \cdot e^{-K \cdot X} + Plateau$ *starting at X = 2 minutes. Data points were plotted as n = 2 with error bars representing ±SEM.* ***D*** *Use of higher concentrations of substrate (1:50) can affect the sensitivity of the assay**.** The fold-gain luminescence signal of anti-rituximab sensor (L-R/S-R) in response to increasing concentrations of rituximab, using varying dilutions of the NanoGlo substrate. Luminescence measurements were taken 2 minutes after substrate addition. Sigmoidal, 4 parameter logistic (4PL) fits were used. Data points were plotted as n = 3, error bars represent ±SEM.* ***E*** *Signal decay is more rapid at lower concentration of substrate (1:2000). Change in fold-gain luminescence signal of anti-rituximab sensor (L-R/S-R) in response to 10 nM rituximab, plotted against time for four dilutions of NanoGlo substrate. RLU readings were taken every 2 minutes from 0-30 minutes post substrate incubation. Non-linear regression curves were fit using a one phase exponential decay equation* $Y = Span \cdot e^{-K \cdot X} + Plateau$ *starting at X = 2 minutes. Data points were plotted as n = 3 with error bars representing ±SEM.* ***F*** *The shaking incubation time does not impact the sensitivity of the assay. Fold gain data from NanoBiT assays performed on the selected optimal LgBiT / SmBiT101 pair for rituximab (L-R/S-R) with varying shaking incubation time at* 25°*C**.** Measurements taken 2 minutes after substrate incubation. Sigmoidal, 4 parameter logistic (4PL) fits were used. Data points were plotted as n = 1.* ***G*** *L-R/S-R is functional in up to 10% pooled human serum. The fold-gain luminescence signal of anti-rituximab sensor (L-R/S-R) in response to increasing concentrations of rituximab, in varying dilutions of pooled human serum. Luminescence measurements were taken 2 minutes after substrate addition. Sigmoidal, 4 parameter logistic (4PL) fits were used. Data points were plotted as n = 3 (0%, 0.1%, 0.4%, 1%) or n = 2 (5%, 50%), error bars represent ±SEM. Unless otherwise specified, these assays were performed with 30 min. incubation between Affimer-constructs and TmAb, followed by addition of 1:1000 NanoGlo and bioluminescence read after 2 min.*

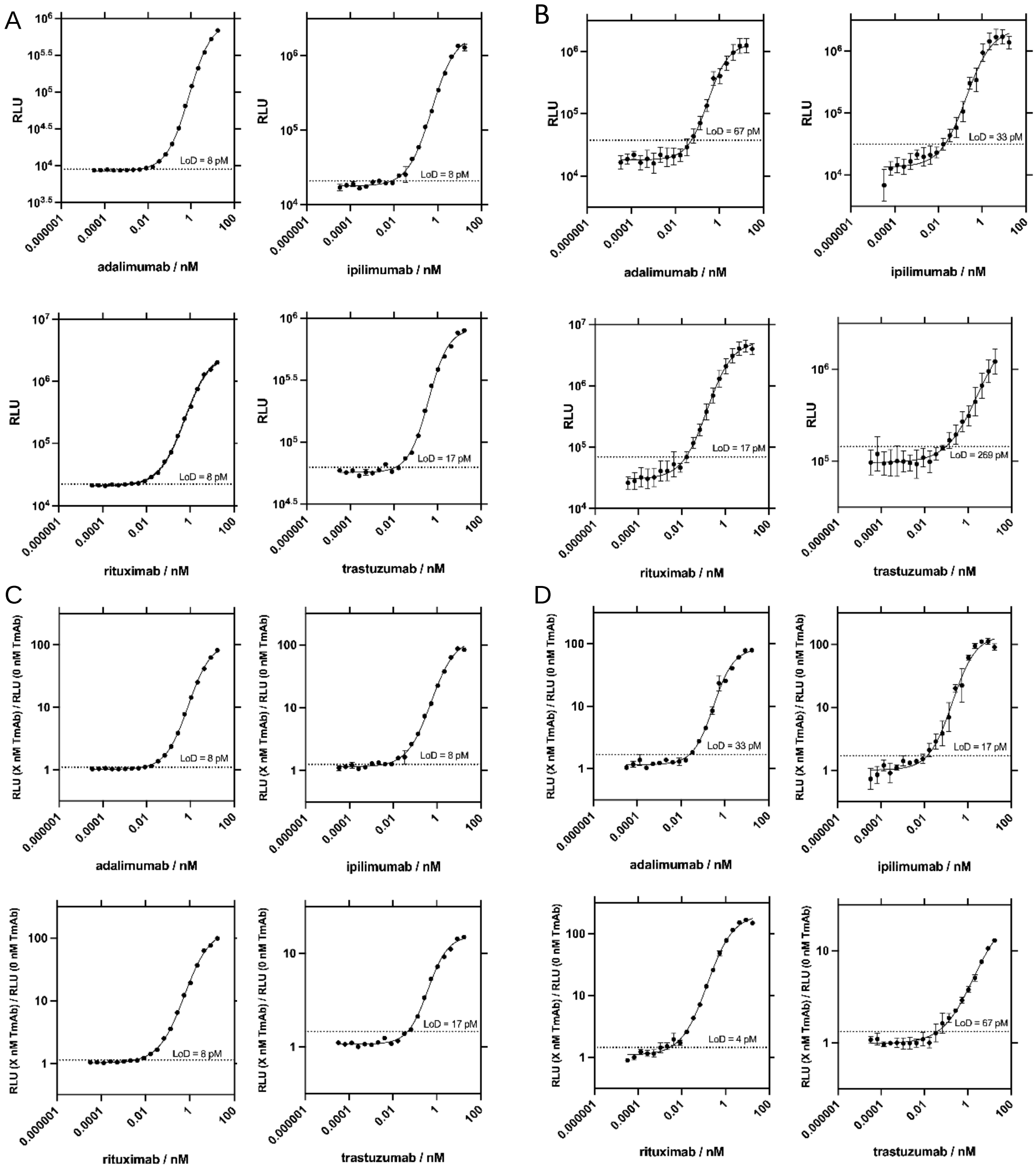

Figure S5. Standard curves of all four TmAb sensor pairs. *Data on all sensors at 2 nM in response to increasing concentrations of their respective TmAb (0.005 – 2500 ng mL^{-1}). Assays were performed in 0.1% pooled human serum, incubated for 5 minutes shaking at 25°C, and RLU measurements taken after 2 minutes. Standard curves were interpolated for all four sensors* ***A & C*** *n = 3, performed on the same day with the same reagents.* ***B & D*** *N = 3, performed on separate days, using fresh reagents. Data points were plotted as raw RLU data (**A & B**) or fold gain from 0 analyte blank measurements (**C & D**), error bars represent ±SEM. Limit of Detection (LoD) values indicated with dashed line. Interpolation of standard curves were completed using Prism 9 GraphPad software.*

Table S1. The reported limit of detection, dilution factor and timeframe to result of commercially available ELISA kits for the detection of rituximab, adalimumab, ipilimumab and trastuzumab in human serum. Reported performance of LUMinescent AntiBody Sensor (LUMABS) for the detection of trastuzumab[1].

TmAb	*Commercial ELISA*	*Biosensor*	*LoD*	*Dilution Factor*	*Time*
Rituximab	ab237640 (Abcam)		~ 31 pM	1000 x	~2.5 hrs
Adalimumab	ab237641 (Abcam)		~ 68 pM	10 x	~2.5 hrs
Trastuzumab	ab237645 (Abcam)		~ 75 pM	1000 x	~2.5 hrs
Ipilimumab	ab237653 (Abcam)		~ 67 pM	300 x	~2.5 hrs
Trastuzumab		LUMABS	~303 nM	N/A	~2.5 hrs

DNA and Protein Sequences

Within the sequence, the Affimer variable region sequences are denoted as XXX. The sequences and plasmids will be shared with any academic who does not have a commercial interest, under an MTA.

LgBit-GSG$_7$-AffX

Protein

MAASVFTLEDFVGDWEQTAAYNLDQVLDQGGVSSLLQNLAVSVTPIQRIVRSGENALKIDIHVIIPYEGLSADQM
AQIEEVFKVVYPVDDHHFKVILPYGTLVIDGVTPNMLNYFGRPYEGIAVFDGKKITVTGTLWNGNKIIDERLITP
DGSMLFRVTINSAAAGSGGSGGSGGSGGSGGSGGSGTSANSLEIEELARFAVDEHNKKENALLEFVRVVKAKEQX
XXXXXXXXTMYYLTLEAKDGGKKKLYEAKVWVKXXXXXXXXXNFKELQEFKPVVDLEHHHHHH

DNA

ATGGCGGCTAGCGTTTTTACCCTGGAAGATTTCGTGGGCGACTGGGAACAGACCGCGGCGTACAACCTGGACCAA
GTGCTGGATCAAGGTGGCGTGAGCAGCCTGCTGCAGAACCTGGCGGTGAGCGTTACCCCGATCCAACGTATTGTT
CGTAGCGGCGAGAACGCGCTGAAGATCGACATTCACGTGATCATTCCGTACGAAGGCCTGAGCGCGGATCAGATG
GCGCAAATCGAGGAAGTGTTCAAGGTGGTTTACCCGGTTGACGATCACCACTTTAAAGTGATCCTGCCGTATGGT
ACCCTGGTGATTGACGGCGTTACCCCGAACATGCTGAACTACTTCGGTCGTCCGTATGAGGGCATCGCGGTTTTT
GATGGTAAGAAAATTACCGTGACCGGTACCCTGTGGAACGGCAACAAAATTATTGATGAGCGCCTGATTACCCCG
GACGGCAGCATGCTGTTTCGTGTGACCATTAATAGCGCGGCCGCTGGGTCCGGCGGTTCAGGCGGCTCTGGTGGC
TCCGGTGGGTCAGGTGGTTCTGGCGGGTCTGGCACTAGTGCAAACTCCCTGGAAATCGAAGAACTGGCTCGTTTC
GCTGTTGACGAACACAACAAAAAAGAAAACGCTCTGCTGGAATTCGTTCGTGTTGTTAAAGCGAAAGAACAGXXX
XXXXXXXXXXXXXXXXXXXXXXXXACCATGTACTACCTGACCCTGGAAGCTAAAGACGGTGGTAAAAAGAAACTG
TACGAAGCGAAAGTTTGGGTTAAAXXXXXXXXXXXXXXXXXXXXXXXXAACTTCAAAGAACTGCAGGAGTTCAAA
CCAGTAGTCGACCTCGAGCACCACCACCACCACCAC

AffX-GSG$_7$-LgBit

Protein

MAASNSLEIEELARFAVDEHNKKENALLEFVRVVKAKEQXXXXXXXXXTMYYLTLEAKDGGKKKLYEAKVWVKXX
XXXXXXXNFKELQEFKPVAAAGSGGSGGSGGSGGSGGSGGSGTSVFTLEDFVGDWEQTAAYNLDQVLEQGGVSSL
LQNLAVSVTPIQRIVRSGENALKIDIHVIIPYEGLSADQMAQIEEVFKVVYPVDDHHFKVILPYGTLVIDGVTPN
MLNYFGRPYEGIAVFDGKKITVTGTLWNGNKIIDERLITPDGSMLFRVTINSVDLEHHHHHH

DNA

ATGGCGGCTAGCAACTCCCTGGAAATCGAAGAACTGGCTCGTTTCGCTGTTGACGAACACAACAAAAAAGAAAAC
GCTCTGCTGGAATTCGTTCGTGTTGTTAAAGCGAAAGAACAGXXXXXXXXXXXXXXXXXXXXXXXXXXXACCATG
TACTACCTGACCCTGGAAGCTAAAGACGGTGGTAAAAAGAAACTGTACGAAGCGAAAGTTTGGGTTAAAXXXXXX
XXXXXXXXXXXXXXXXXXAACTTCAAAGAACTGCAGGAGTTCAAACCAGTAGCGGCCGCTGGGTCCGGCGGTTCA
GGCGGCTCTGGTGGCTCCGGTGGGTCAGGTGGTTCTGGCGGGTCTGGCACTAGTGTTTTTACCCTGGAAGATTTC
GTGGGCGACTGGGAACAGACCGCGGCGTACAACCTGGACCAAGTGCTGGAACAAGGTGGCGTGAGCAGCCTGCTG
CAGAACCTGGCGGTGAGCGTTACCCCGATCCAACGTATTGTTCGTAGCGGCGAGAACGCGCTGAAGATCGACATT
CACGTGATCATTCCGTACGAAGGCCTGAGCGCGGATCAGATGGCGCAAATCGAGGAAGTGTTCAAGGTGGTTTAC
CCGGTTGACGATCACCACTTTAAAGTGATCCTGCCGTATGGTACCCTGGTGATTGACGGCGTTACCCCGAACATG
CTGAACTACTTCGGTCGTCCGTATGAGGGCATCGCGGTTTTTGATGGTAAGAAAATTACCGTGACCGGTACCCTG
TGGAACGGCAACAAAATTATTGATGAGCGCCTGATTACCCCGGACGGCAGCATGCTGTTTCGTGTGACCATTAAT
AGCGTCGACCTCGAGCACCACCACCACCACCAT

SmBit101-GSG$_7$-AffX
Protein
MAASVTGYRLFEKESAAAGSGGSGGSGGSGGSGGSGGSGTSANSLEIEELARFAVDEHNKKENALLEFVRVVKAK
EQXXXXXXXXXTMYYLTLEAKDGGKKKLYEAKVWVKXXXXXXXXXNFKELQEFKPVVDLEHHHHHH
DNA
ATGGCGGCTAGCGTAACAGGATATAGGCTATTTGAAAAAGAGAGCGCGGCCGCTGGGTCCGGCGGTTCAGGCGGC
TCTGGTGGCTCCGGTGGGTCAGGTGGTTCTGGCGGGTCTGGCACTAGTGCAAACTCCCTGGAAATCGAAGAACTG
GCTCGTTTCGCTGTTGACGAACACAACAAAAAAGAAAACGCTCTGCTGGAATTCGTTCGTGTTGTTAAAGCGAAA
GAACAGXXXXXXXXXXXXXXXXXXXXXXXXXXXACCATGTACTACCTGACCCTGGAAGCTAAAGACGGTGGTAAA
AAGAAACTGTACGAAGCGAAAGTTTGGGTTAAAXXXXXXXXXXXXXXXXXXXXXXXXAACTTCAAAGAACTGCAG
GAGTTCAAACCAGTAGTCGACCTCGAGCACCACCACCACCACCAC

AffX-GSG$_7$-SmBit101
Protein
MAASNSLEIEELARFAVDEHNKKENALLEFVRVVKAKEQXXXXXXXXXTMYYLTLEAKDGGKKKLYEAKVWVKXX
XXXXXXXNFKELQEFKPVAAAGSGGSGGSGGSGGSGGSGGSGTSVTGYRLFEKESVDLEHHHHHH
DNA
ATGGCGGCTAGCAACTCCCTGGAAATCGAAGAACTGGCTCGTTTCGCTGTTGACGAACACAACAAAAAAGAAAAC
GCTCTGCTGGAATTCGTTCGTGTTGTTAAAGCGAAAGAACAGXXXXXXXXXXXXXXXXXXXXXXXXXXXACCATG
TACTACCTGACCCTGGAAGCTAAAGACGGTGGTAAAAAGAAACTGTACGAAGCGAAAGTTTGGGTTAAAXXXXXX
XXXXXXXXXXXXXXXXXXAACTTCAAAGAACTGCAGGAGTTCAAACCAGTAGCGGCCGCTGGGTCCGGCGGTTCA
GGCGGCTCTGGTGGCTCCGGTGGGTCAGGTGGTTCTGGCGGGTCTGGCACTAGTGTAACAGGATATAGGCTATTT
GAAAAAGAGAGCGTCGACCTCGAGCACCACCACCACCACCAC

Tables of Primers

Table S2. Primers used for Affimer amplification

Primer	Sequence
NheI-Aff-f	ATGCGCTAGCAACTCCCTGGAAATCGAAGAACTG
NotI-Aff-r	TAATGCGGCCGCTACTGGTTTGAACTCCTGCAGTTCTTTG
SpeI-Aff-f	AACGACTAGTGCAAACTCCCTGGAAATCGAAGAACTG
SalI-Aff-r	TAATGTCGACTACTGGTTTGAACTCCTGCAGTTCTTTG

Table S3. Primers used for LgBiT amplification

Primer	Sequence
LgBiT-NheI-f	TAATGCTAGCGTTTTTACCCTGGAAGATTTCG
LgBiT-NotI-r	TTATGCGGCCGCGCTATTAATGGTCACACGAAAC
LgBiT-SpeI-f	TAAAACTAGTGTTTTTACCCTGGAAGATTTCG
LgBiT-SalI-r	TAATGTCGACGCTATTAATGGTCACACGAAAC

Table S4. Primers used to generate SmBiT (101)

Primer	Sequence
SmBiT101-NheI-f	TAATGCTAGCGTAACAGGATATAGGCTATTTGAAAAAGAGAGC
SmBiT101-NotI-r	TTATGCGGCCGCGCTCTCTTTTTCAAATAGCCTATATCCTGTTAC
SmBiT101-SpeI-f	TAAAACTAGTGTAACAGGATATAGGCTATTTGAAAAAGAGAGC
SmBiT101-SalI-r	TAATGTCGACGCTCTCTTTTTCAAATAGCCTATATCCTGTTAC

Table S5. Primers used to generate GSG_7 linker

Primer	Sequence
NotI-GSG_7-f	TTATGCGGCCGCTGGGTCCGGCGGTTCAGGCGGCTCTGGTGGCTCCGGTGGGTCAGGT
SpeI-GSG_7-r	TAAAACTAGTGCCAGACCCGCCAGAACCACCTGACCCACCGGAGCCACCAGAGCC

1. M. van Rosmalen, Y. Ni, D. F. M. Vervoort, R. Arts, S. K. J. Ludwig and M. Merkx, *Analytical Chemistry*, 2018, **90**, 3592-3599
